# Supplementary material for: Macro-connectomics and microstructure predict dynamic plasticity patterns in the non-human primate brain
Source: eLife. 2018 Nov 21;7:e34354. doi: 10.7554/eLife.34354 (PMC6249000; doi:10.7554/eLife.34354)
Supplement: Transparent reporting form [file elife-34354-transrepform.pdf]

eLIFE
1st Floor
24 Hills Road
Cambridge CB2 1JP, UK
P 01223 855340
W elifesciences.org
T @elife

# *eLife's* transparent reporting form

We encourage authors to provide detailed information *within their submission* to facilitate the interpretation and replication of experiments. Authors can upload supporting documentation to indicate the use of appropriate reporting guidelines for health-related research (see EQUATOR Network), life science research (see the BioSharing Information Resource), or the ARRIVE guidelines for reporting work involving animal research. Where applicable, authors should refer to any relevant reporting standards documents in this form.

If you have any questions, please consult our Journal Policies and/or contact us: editorial@elifesciences.org.

**Sample-size estimation**

- You should state whether an appropriate sample size was computed when the study was being designed
- You should state the statistical method of sample size computation and any required assumptions
- If no explicit power analysis was used, you should describe how you decided what sample (replicate) size (number) to use

Please outline where this information can be found within the submission (e.g., sections or figure legends), or explain why this information doesn't apply to your submission:

The number of animals used for this study were determined by the behavioral study for which they were originally assigned, and the sample size determined based on a power analysis designed to elicit behavioral effects. The details are available in the behavioral study, which is currently available as a preprint: Froudist-Walsh, S., Browning, P.G.F., Croxson, P.L., Murphy, K.L., Shamy, J.L., Veuthey, T.L., Wilson, C.R.E., and Baxter, M.G. (2018). The rhesus monkey hippocampus critically contributes to scene memory retrieval, but not new learning. BioRxiv 288407. doi: https://doi.org/10.1101/288407

**Replicates**

- You should report how often each experiment was performed
- You should include a definition of biological versus technical replication
- The data obtained should be provided and sufficient information should be provided to indicate the number of independent biological and/or technical replicates
- If you encountered any outliers, you should describe how these were handled
- Criteria for exclusion/inclusion of data should be clearly stated
- High-throughput sequence data should be uploaded before submission, with a private link for reviewers provided (these are available from both GEO and ArrayExpress)

Please outline where this information can be found within the submission (e.g., sections or figure legends), or explain why this information doesn't apply to your submission:

eLife Sciences Publications, Ltd is a limited liability non-profit non-stock corporation incorporated in the State of Delaware, USA, with company number 5030732, and is registered in the UK with company number FC030576 and branch number BR015634 at the address 1st Floor, 24 Hills Road, Cambridge CB2 1JP | August 2014

Each experiment was performed once, because this was a study using macaque monkeys, the use of which strict guidelines exist for the number of procedures that can be carried out on each animal. The number of animals and scans (which were carried out under anaesthesia) was therefore kept to a minimum. Scans for which data were not of sufficient quality (high noise or very low contrast) were excluded from the analysis. This is described in the Methods section.

**Statistical reporting**

- Statistical analysis methods should be described and justified
- Raw data should be presented in figures whenever informative to do so (typically when N per group is less than 10)
- For each experiment, you should identify the statistical tests used, exact values of N, definitions of center, methods of multiple test correction, and dispersion and precision measures (e.g., mean, median, SD, SEM, confidence intervals; and, for the major substantive results, a measure of effect size (e.g., Pearson's r, Cohen's d)
- Report exact p-values wherever possible alongside the summary statistics and 95% confidence intervals. These should be reported for all key questions and not only when the p-value is less than 0.05.

Please outline where this information can be found within the submission (e.g., sections or figure legends), or explain why this information doesn't apply to your submission:

Statistical analysis methods and criteria are clearly reported in the Methods section for all analyses carried out.

(For large datasets, or papers with a very large number of statistical tests, you may upload a single table file with tests, Ns, etc., with reference to sections in the manuscript.)

**Group allocation**

- Indicate how samples were allocated into experimental groups (in the case of clinical studies, please specify allocation to treatment method); if randomization was used, please also state if restricted randomization was applied
- Indicate if masking was used during group allocation, data collection and/or data analysis

Please outline where this information can be found within the submission (e.g., sections or figure legends), or explain why this information doesn't apply to your submission:

Animals were pseudorandomly assigned to lesion or control groups based on their pre-operative behavioural performance, to assure no statistical difference in behavioural performance prior to the lesion. This is outlined in the behavioural study preprint: Froudist-Walsh, S., Browning, P.G.F., Croxson, P.L., Murphy, K.L., Shamy, J.L., Veuthey, T.L., Wilson, C.R.E., and Baxter, M.G. (2018). The rhesus monkey hippocampus critically contributes to scene memory retrieval, but not new learning. BioRxiv 288407. doi: https://doi.org/10.1101/288407. Masking was not used during data collection or analysis as the data are MRI data and each dataset was treated the same way for analysis regardless of group.

**Additional data files ("source data")**

- We encourage you to upload relevant additional data files, such as numerical data that are represented as a graph in a figure, or as a summary table
- Where provided, these should be in the most useful format, and they can be uploaded as "Source data" files linked to a main figure or table
- Include model definition files including the full list of parameters used
- Include code used for data analysis (e.g., R, MatLab)
- Avoid stating that data files are "available upon request"

Please indicate the figures or tables for which source data files have been provided:

We are happy to provide the code and data used for the analysis. We are still working on commenting the code so will arrange for this to be provided as soon as possible; definitely upon publication.
